# 436. U-shaped-aggressiveness of SARS-CoV-2: Period Between Initial Symptoms and Clinical Progression to COVID-19 Suspicion. A Population-Based Cohort Study

**DOI:** 10.1093/ofid/ofab466.635

**Published:** 2021-12-04

**Authors:** Bruno G Buitano, Dan Morgenstern, Juan Talavera, Andrea Zaldívar, Mercedes Martínez

**Affiliations:** 1 Universidad Anáhuac México, Mexico City, Distrito Federal, Mexico; 2 ABC Hospital México, Mexico City, Distrito Federal, Mexico

## Abstract

**Background:**

Until now, studies have been focused on patient-centered risk factors, while SARS-CoV-2 aggressiveness has been established as causing 20% of severe and critical patients. However, there are still many unanswered questions concerning the clinical aggressiveness behavior of SARS-CoV-2. This study focuses on progression of symptoms as a marker of such aggressiveness, using the Period between initial symptoms and clinical progression to COVID-19 suspicion (PISYCS) to determine the risk of severe disease and mortality.

**Methods:**

Historic cohort study of Mexican patients. Data from January-April 2020 were provided by the Health Ministry. Setting: Population-based. Patients registered in the Epidemiologic Surveillance System in Mexico. Participants were subjects who sought medical attention for clinical suspicion of COVID-19. All patients were subjected to RT-PCR testing for SARS-CoV-2. We measured the Period between initial symptoms and clinical progression to COVID-19 suspicion (PISYCS) and compared it to the primary outcomes (mortality and pneumonia)

**Results:**

65,500 patients were included. Reported fatalities and pneumonia were 2176 (3.32%), and 11568 (17.66%), respectively. According to the PISYCS, patients were distributed as follows: 14.89% in < 24 hours, 43.25% between 1–3 days, 31.87% between 4–7 days and 9.97% > 7 days. The distribution for mortality and pneumonia was 5.2% and 22.5% in < 24 hours, 2.5% and 14% between 1–3 days, 3.6% and 19.5% between 4–7 days, 4.1% and 20.6% > 7 days, respectively (p< 0.001). Adjusted-risk of mortality was (OR [95% CI], p-value): < 24 hours = 1.75 [1.55–1.98], p< 0.001; 1–3 days = 1 (reference value); 4–7 days = 1.53 [1.37–1.70], p< 0.001; > 7 days = 1.67 [1.44–1.94], p< 0.001. For pneumonia: < 24 hours = 1.49 [1.39–1.58], p< 0.001; 1–3 days = 1; 4–7 days = 1.48 [1.41–1.56], p< 0.001; > 7 days = 1.57 [1.46–1.69], p< 0.001.

Risk of Mortality vs. PISYCS

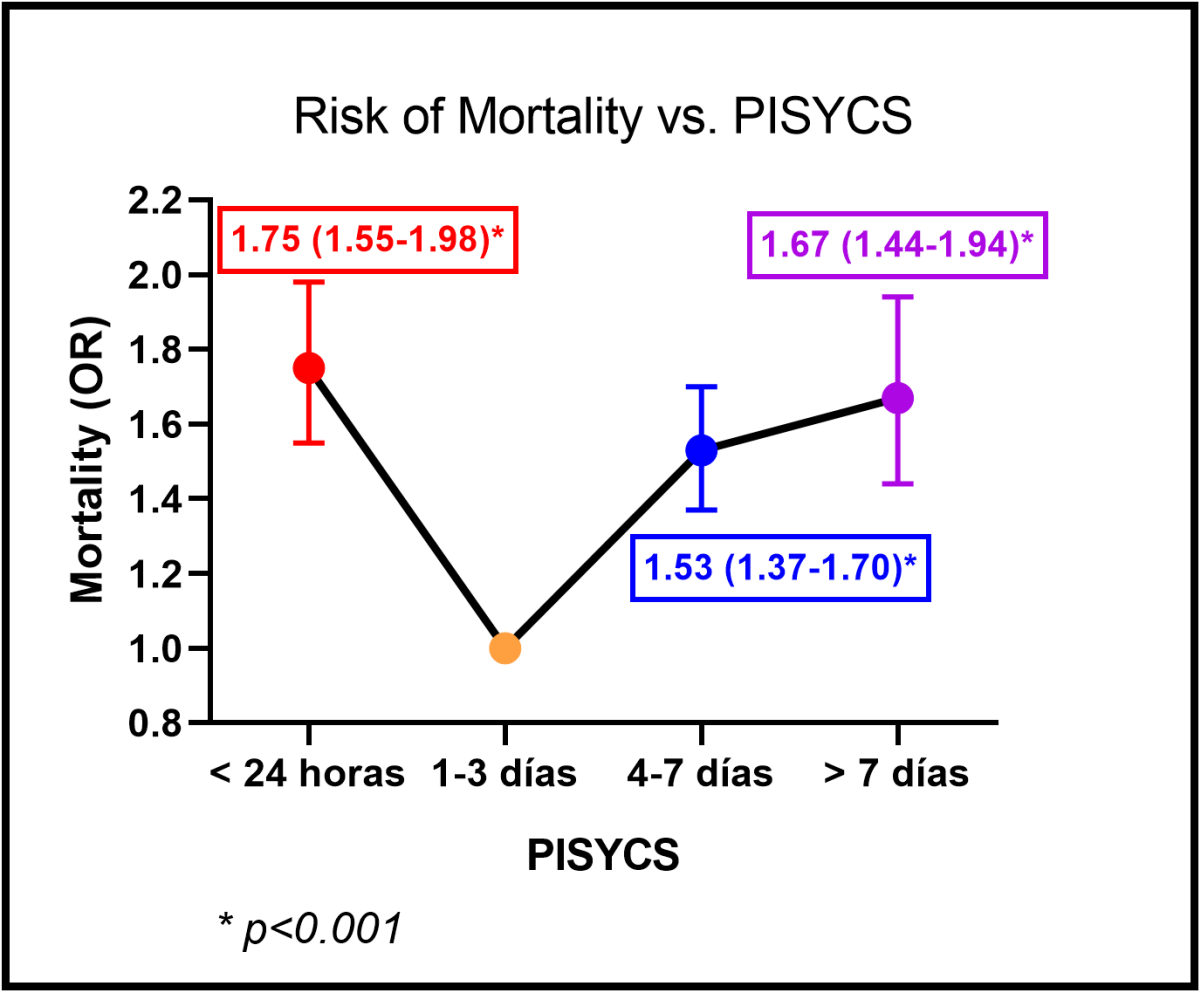

Logistic regression anlaysis of mortality based on PISYCS. Note that risk of mortality is significantly higher when PISYCS is > 24 hours and < 7 days

Risk of Pneumonia vs. PISYCS

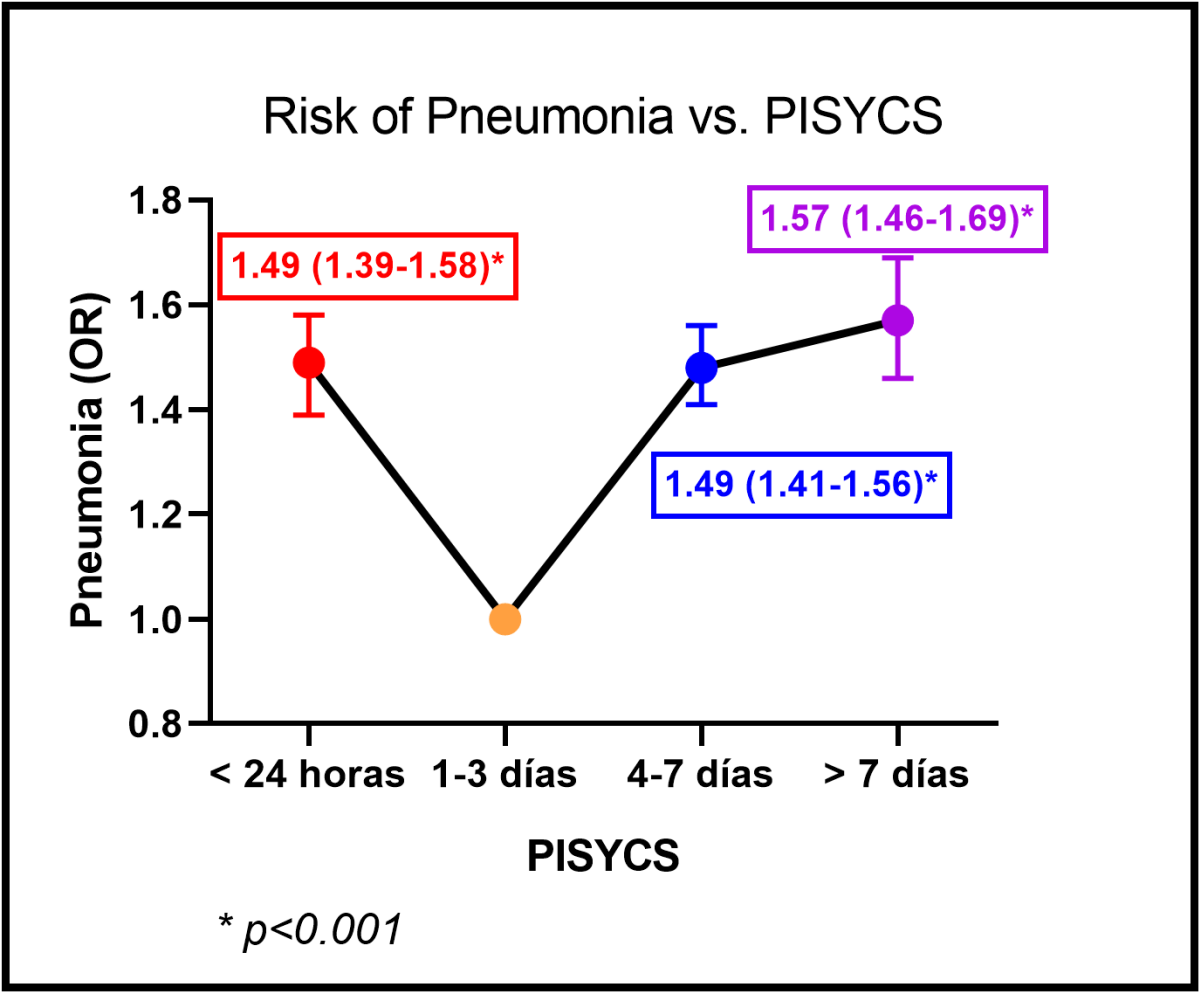

Logistic regression anlaysis of developing pneumonia based on PISYCS. Note that risk of pneumonia is significantly higher when PISYCS is > 24 hours and < 7 days.

**Conclusion:**

The PISYCS shows a U-shaped SARS-CoV-2 aggressiveness pattern. Further studies are needed to corroborate the time-related pathophysiology behind these findings and possibly justify use of PISYCS as an initial evaluation tool and therapies/monitoring in high-risk patients.

**Disclosures:**

**All Authors**: No reported disclosures

